# Comparative outcomes of phenol therapy and Karydakis flap in pilonidal sinus disease: A retrospective cohort study with 1-year follow-up

**DOI:** 10.1097/MD.0000000000050616

**Published:** 2026-09-04

**Authors:** İgbal Osmanov, Bariş Hazir, Uğur Ekici

**Affiliations:** a Department of General Surgery, Ufuk University Faculty of Medicine, Ankara, Türkiye.

**Keywords:** Karydakis flap, phenol therapy, return-to-work time, sacrococcygeal pilonidal sinus, wound healing time

## Abstract

Sacrococcygeal pilonidal sinus disease (PSD) is commonly treated by surgical excision with flap reconstruction. However, minimally invasive treatment modalities based on chemical ablation have gained increasing interest because PSD is not considered a neoplastic disease. Among these techniques, phenol therapy (PT) has emerged as an effective outpatient treatment associated with rapid recovery, low morbidity, and acceptable recurrence rates. To compare the 1-year clinical outcomes of PT and the Karydakis flap (KF) procedure in patients with primary sacrococcygeal PSD. This retrospective cohort study included 125 consecutive patients treated between January 2024 and January 2025. Seventy-seven patients underwent the KF procedure, whereas 48 patients received PT. Demographic characteristics, wound healing time, return-to-work time, postoperative complications, and recurrence rates were evaluated. Patients completed at least 1 year of follow-up through scheduled outpatient visits and structured telephone interviews. The mean age of the study population was 23.68 ± 8.21 years. No significant differences were observed between the groups regarding age, sex, body mass index, family history, prolonged sitting occupation, or history of preoperative abscess drainage (all *P* > .05). Wound healing time was significantly shorter in the phenol group than in the KF group (15.4 ± 8.4 vs 36.1 ± 7.6 days, *P* < .005). Similarly, the return-to-work time was significantly shorter following PT. At 1-year follow-up, recurrence occurred in 6 patients (12.5%) in the phenol group and 7 patients (9.1%) in the KF group, with no statistically significant difference between the groups (*P* = .09). PT was associated with faster wound healing and earlier return to work, while the 1-year recurrence rate did not differ significantly from that of the KF procedure. It may therefore represent a practical minimally invasive treatment alternative for carefully selected patients with primary sacrococcygeal PSD.

## 1. Introduction

Pilonidal sinus disease (PSD) is a chronic inflammatory condition of the subcutaneous tissue that most commonly occurs in the sacrococcygeal region. It primarily affects young adults, particularly after puberty when pilosebaceous gland activity increases. The disease was first described by Hodges in 1880.^[1]^ PSD is characterized by 1 or more midline sinus openings within the natal cleft and is frequently associated with chronic infection.^[2]^

The etiology of PSD has long been debated, with both congenital and acquired mechanisms being proposed. However, current evidence strongly supports an acquired origin.^[3]^ Several predisposing factors have been associated with the development of PSD, including male sex, obesity, excessive body hair, oily skin, prolonged sitting, and poor local hygiene.^[4]^

Various treatment options have been described for PSD, including curettage, phenol therapy (PT), laser therapy, and surgical procedures. Although surgical treatment remains the standard approach for many patients, minimally invasive techniques have gained increasing attention because they preserve healthy tissue, can be performed in an outpatient setting, and facilitate earlier recovery.^[5]^ Among surgical techniques, the Karydakis flap (KF) is one of the most widely accepted off-midline flap procedures because of its favorable postoperative outcomes and low recurrence rates.

Among minimally invasive approaches, PT has emerged as an effective alternative to surgery. Previous studies have demonstrated that PT is associated with shorter wound healing time, earlier return to work, less postoperative pain, and acceptable recurrence rates.^[6–9]^ Nevertheless, direct comparative evidence between PT and flap procedures remains limited, particularly regarding 1-year clinical outcomes in young and working-age patients.

Therefore, the present study aimed to compare the 1-year clinical outcomes, wound healing time, return-to-work time, postoperative complications, and recurrence rates of patients with primary sacrococcygeal PSD treated with either the KF or PT.

## 2. Materials and methods

### 2.1. Study design and patients

This retrospective cohort study included patients with primary sacrococcygeal PSD who underwent either the KF procedure or PT between January 2024 and January 2025. This single-center study was conducted at an academic center in Ankara, Türkiye. The study was conducted and reported in accordance with the Strengthening the Reporting of Observational Studies in Epidemiology guidelines.

The inclusion criteria were: diagnosis of primary sacrococcygeal PSD, treatment with either KF or PT during the study period, and completion of at least 1 year of follow-up.

The exclusion criteria were: recurrent PSD, previous definitive surgery for PSD, incomplete medical records, and failure to complete 1-year follow-up.

After receiving detailed information regarding both treatment options, including their advantages and disadvantages, patients and/or their legal guardians selected the preferred treatment modality, and written informed consent was obtained before treatment.

A total of 125 patients met the eligibility criteria. Of these, 77 underwent the KF procedure and 48 received PT.

### 2.2. Clinical data collection

Medical records were reviewed to obtain demographic and clinical data, including age, sex, body mass index (BMI), history of previous abscess drainage, prolonged sitting occupation, wound healing time, return-to-work time, postoperative complications, and recurrence. Follow-up data were obtained through scheduled outpatient visits and structured telephone interviews. During telephone interviews, patients were specifically questioned regarding wound discharge, pain, development of a new sinus opening, additional treatment, recurrence, and return-to-work time. Patients with suspected recurrence were invited for clinical examination.

All patients presenting with purulent discharge received oral amoxicillin/clavulanic acid (1 g twice daily) for 1 week before definitive treatment. Patients presenting with a pilonidal abscess underwent incision and drainage followed by oral amoxicillin/clavulanic acid (1 g twice daily) and metronidazole (500 mg twice daily). Definitive treatment was performed 7 to 10 days after drainage.

### 2.3. PT technique

PT was performed under local anesthesia in an outpatient setting. Following local anesthesia, the sinus openings were cannulated using a stylet. A 0.5 to 1 cm incision, including the sinus opening closest to the cyst, was made to access the cavity. The sinus tract was thoroughly curetted to remove hair, debris, and granulation tissue, ensuring adequate penetration of phenol into the cavity.

Before phenol application, nitrofurazone ointment was applied to the surrounding skin to protect it from the caustic effect of phenol. Subsequently, 80% liquid phenol was applied to the entire cyst wall. In patients with lateral sinus openings, phenol was also instilled through the external opening. Excess phenol was then drained from the cavity.

**Figure 1.**
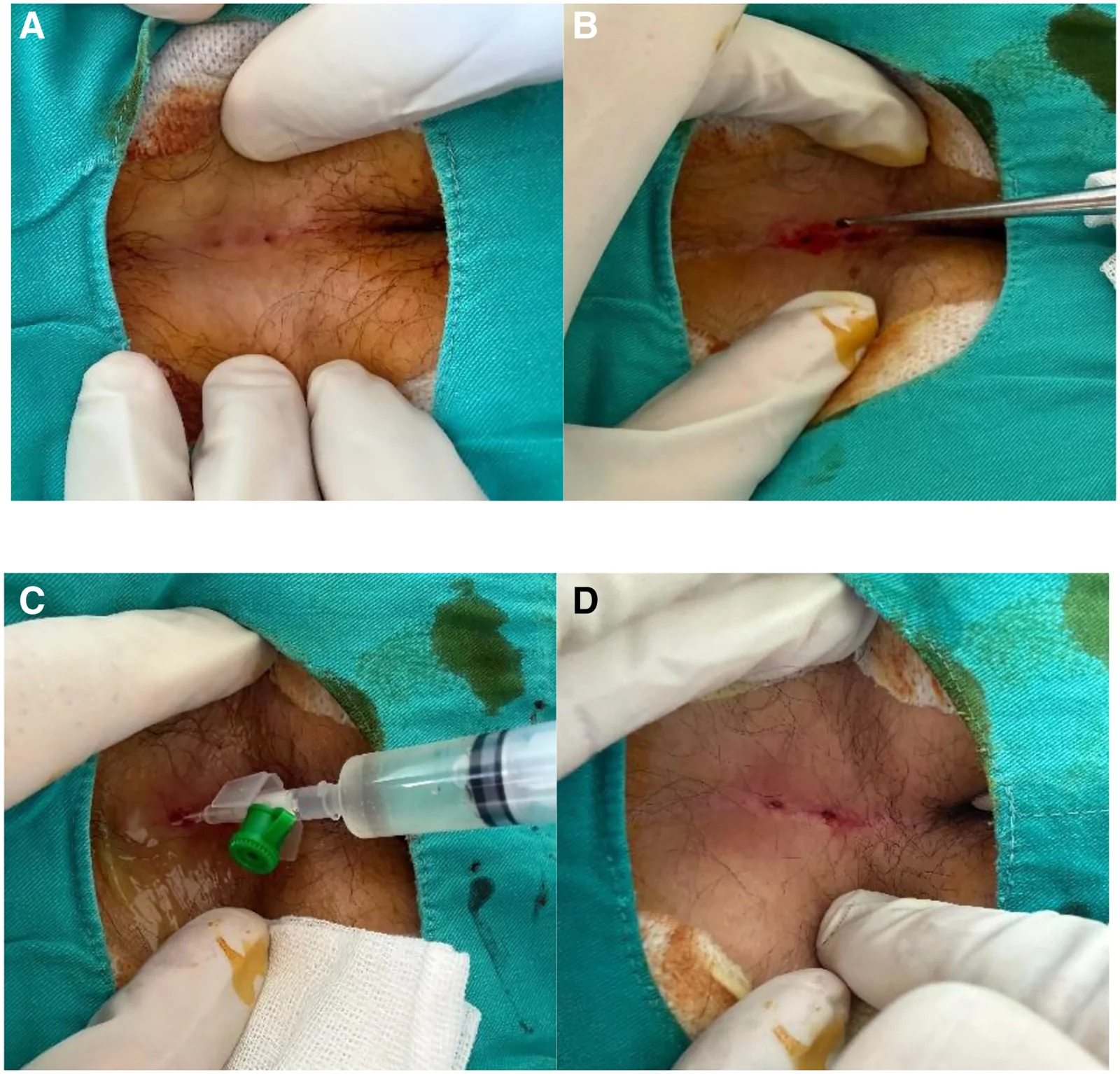
Phenol application technique for pilonidal sinus. (A) Sinus opening, (B) curettage, (C) application of 80% liquid phenol after protection of the surrounding skin with nitrofurazone ointment, (D) final view.

Patients were reevaluated 15 days after the procedure. If complete healing had not been achieved, a second phenol application was performed. Treatment success was defined as complete epithelialization of the sinus tract without discharge or signs of infection (Fig. 1).

### 2.4. KF technique

The KF procedure was performed under spinal anesthesia in the operating room. With the patient in the jackknife position and the gluteal region laterally retracted, an asymmetric biconcave elliptical incision was made to include all sinus openings. The cyst and all sinus tracts were completely excised.

**Figure 2.**
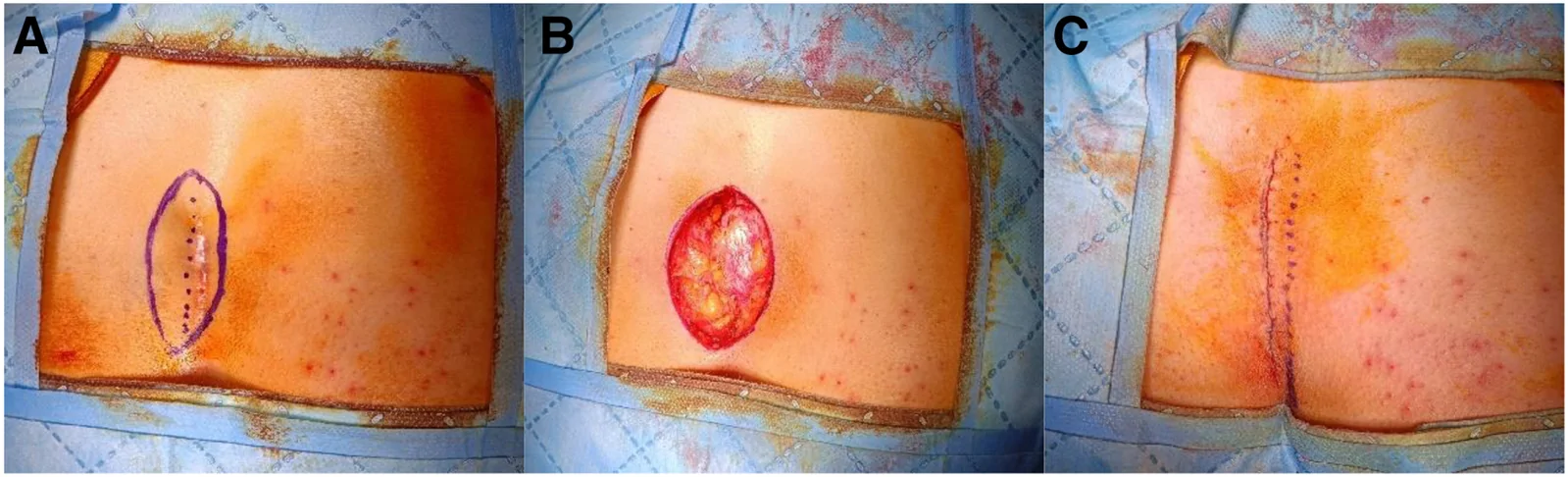
KF procedure for sacrococcygeal pilonidal sinus. (A) Preoperative mapping and marking of the elliptical incision line, (B) excision of the pilonidal sinus and surrounding tissue, (C) final closure with lateralization of the natal cleft. KF = Karydakis flap.

A flap approximately 1 cm in depth and 2 cm medial to the incision line was prepared using electrocautery. The flap was fixed to the presacral fascia using absorbable 2/0 sutures. A closed-suction drain was placed when considered necessary by the operating surgeon and, when used, was routinely removed on the first postoperative day. A second layer of absorbable sutures was placed between the undersurface of the flap and the lateral adipose tissue. Skin closure was performed using mattress or intracutaneous sutures (Fig. 2).

Routine wound dressings were discontinued on the second postoperative day. Routine postoperative analgesia consisted of oral paracetamol (500 mg twice daily) prescribed for 3 days.

### 2.5. Follow-up and outcome assessment

Patients were routinely followed through scheduled outpatient visits. In addition, structured telephone interviews were conducted to assess wound healing, return-to-work time, recurrence, and the need for additional treatment. Patients reporting symptoms suggestive of recurrence were invited for clinical examination.

A completed 1-year follow-up was defined as the availability of clinical follow-up and/or successful telephone contact at least 12 months after treatment. Patients who could not be contacted or did not complete the 1-year follow-up were excluded from the study.

Recurrence was defined as the development of a new sinus opening, persistent or recurrent discharge, or the need for additional treatment after complete initial wound healing.

### 2.6. Statistical analysis

Statistical analyses were performed using IBM SPSS Statistics for Windows, Version 24.0 (IBM Corp.). The normality of continuous variables was assessed using the Shapiro–Wilk test. Normally distributed continuous variables were compared using Student *t* test, whereas the Mann–Whitney *U* test was used for variables that did not follow a normal distribution. Categorical variables were compared using the chi-square test.

Continuous variables are presented as mean ± standard deviation, and categorical variables are presented as frequencies and percentages. All statistical tests were 2-tailed, and a *P* value < .05 was considered statistically significant.

The statistical analyses were independently reviewed by a biomedical statistician.

A post hoc power assessment was performed for the comparison of recurrence rates between the PT and KF groups. Based on the observed recurrence rates of 12.5% in the PT group and 9.1% in the KF group, with a 2-sided alpha level of 0.05 and the current sample sizes, the estimated statistical power was approximately 9%. This low statistical power indicates that the study may have been underpowered to detect a small difference in recurrence rates and supports the possibility of a type II error.

### 2.7. Ethics statement

The study was conducted in accordance with the ethical principles of the Declaration of Helsinki and was approved by the institutional non-interventional clinical research ethics committee (Decision No. 25.07.25.05/01). Written informed consent was obtained from all patients and/or their legal guardians before treatment.

## 3. Results

A total of 125 patients met the study eligibility criteria, including 77 patients in the KF group and 48 patients in the PT group. In the KF group, 55 patients (71.4%) were male and 22 (28.6%) were female, whereas the PT group included 33 male patients (68.8%) and 15 female patients (31.2%). The overall mean age of the study population was 23.68 ± 8.21 years, with a median age of 22 years.

There were no statistically significant differences between the 2 groups in terms of age, sex, BMI, family history, prolonged sitting occupation, or history of preoperative abscess drainage (all *P* > .05) (Table 1).

**Table 1:** Patient characteristics according to treatment group.

Variable	PT group (n = 48)	KF group (n = 77)	*P* value
Sex n (%)			
Female	15 (31.2)	22 (28.6)	.37
Male	33 (68.8)	55 (71.4)	
Age, years, mean ± SD (min–max)/median	23.5 ± 5.9 (14–42)/22	24.4 ± 7.0 (15–50)/22	.446
BMI, kg/m^2^, mean ± SD (min–max)/median	25.1 ± 3.6 (16–36)/24.8	25.1 ± 3.1 (15–32)/25.1	.659
Family history, n (%)	18 (37.5)	26 (33.8)	.352
Prolonged sitting occupation, n (%)	21 (43.8)	38 (49.4)	.203
History of preoperative abscess drainage, n (%)	5 (10.4)	10 (13)	.245

BMI = body mass index, KF = Karydakis flap, n = number of patients, PT = phenol therapy, SD = standard deviation.

In the PT group, 31 patients underwent a single phenol application, whereas 17 required 2 treatment sessions. No postoperative complications were observed in the PT group. In the KF group, postoperative seroma developed in 7 patients (9.1%), and surgical site infection occurred in 7 patients (9.1%). Seromas were managed by percutaneous needle aspiration, whereas surgical site infections were treated with local wound care and oral antibiotic therapy. None of the patients required reoperation. According to the Clavien–Dindo classification, seromas requiring percutaneous needle aspiration were classified as grade IIIa, whereas surgical site infections treated with local wound care and oral antibiotic therapy were classified as grade II. No grade IIIb or higher complications occurred. Overall, postoperative complications were significantly more frequent in the KF group than in the PT group (*P* < .001).

Wound healing time was significantly shorter in the PT group than in the KF group (15.4 ± 8.4 vs 36.1 ± 7.6 days, respectively; *P* < .005). Similarly, return-to-work time was significantly shorter in the PT group. The mean return-to-work time was 5.9 ± 2.0 days in the PT group and 25.2 ± 7.0 days in the KF group (*P* < .05).

At the 1-year follow-up, recurrence was observed in 6 patients (12.5%) in the PT group and 7 patients (9.1%) in the KF group. Although the recurrence rate was numerically higher in the PT group, the difference did not reach statistical significance (*P* = .09) (Table 2).

**Table 2:** Comparison of postoperative outcomes between the PT and KF groups.

Variable	PT group (n = 48)	KF group (n = 77)	*P* value
Postoperative complications, n (%)	None	Seroma:7 (9.1) Surgical site infection: 7 (9.1)	< .001*
Wound healing time, d, mean ± SD (min–max)	15.4 ± 8.4 (11–27)	36.1 ± 7.6 (29–48)	< .005*
Return-to-work time, d, mean ± SD (min–max)	5.9 ± 2 (3–7)	25.2 ± 7 (20–34)	< .05*
Recurrence, n (%)	6 (12.5)	7 (9.1)	.09

Seromas were managed by percutaneous needle aspiration and classified as Clavien–Dindo grade IIIa. Surgical site infections were treated with local wound care and oral antibiotic therapy and classified as Clavien–Dindo grade II. No grade IIIb or higher complications occurred.

KF = Karydakis flap, n = number of patients, PT = phenol therapy, SD = standard deviation.

* Statistically significant.

## 4. Discussion

The present study compared the 1-year clinical outcomes of PT and the KF in patients with primary sacrococcygeal PSD. The principal findings were that PT was associated with significantly shorter wound healing and return-to-work times, while the 1-year recurrence rate did not differ significantly between the 2 groups. Although postoperative complications occurred more frequently in the KF group, all complications were managed successfully without the need for reoperation.

The baseline demographic and clinical characteristics were comparable between the PT and KF groups, with no significant differences in age, sex, BMI, family history, prolonged sitting occupation, or history of preoperative abscess drainage. This similarity reduces the potential influence of measured baseline confounding factors and supports the comparison of postoperative outcomes between the 2 treatment modalities.

KF is an established off-midline flap procedure for PSD and has been associated with favorable recurrence rates compared with midline closure techniques. Karydakis originally reported low recurrence rates, and subsequent studies and meta-analyses have supported the effectiveness of off-midline flap procedures in reducing recurrence and improving postoperative outcomes.^[10–18]^ In the present study, the recurrence rate observed in the KF group was consistent with these previous reports, supporting KF as an effective surgical option, particularly in patients for whom flap-based treatment is preferred or required.

PT has been increasingly used as a minimally invasive treatment option for PSD. Previous studies have reported favorable outcomes with phenol-based treatment, including acceptable recurrence rates, shorter recovery time, and reduced postoperative morbidity compared with conventional surgical approaches.^[6–9,19–25]^ The shorter wound healing and return-to-work times observed in the PT group in the present study are consistent with the minimally invasive nature of this treatment. Unlike flap-based surgery, PT does not require wide tissue excision, flap mobilization, or extensive wound closure. This may explain the more rapid postoperative recovery and earlier return to daily activities in the PT group. These advantages are particularly important for young and working-age individuals, in whom early recovery and prompt return to daily life are major treatment goals.

Because phenol is a caustic agent, careful protection of the surrounding skin is essential during application. Safety concerns and regulatory differences regarding phenol use have previously been discussed in the literature.^[26]^ In the present study, the surrounding skin was protected with nitrofurazone ointment before phenol application, and no postoperative complications were observed in the PT group.

Recent studies have also focused on optimizing minimally invasive treatment strategies for PSD. Platelet-rich plasma has been evaluated as an adjunct to crystallized phenol to enhance wound healing, whereas laser-based treatment has been compared with crystallized phenol as another minimally invasive alternative.^[27,28]^ These studies reflect the current trend toward tissue-sparing approaches that aim to reduce postoperative pain, shorten recovery, and maintain acceptable recurrence rates. In this context, the findings of the present study support the role of PT as a practical, minimally invasive option, particularly for patients in whom rapid recovery and early return to work are important treatment priorities.

The recurrence findings should be interpreted with caution. In the present study, recurrence was numerically higher in the PT group than in the KF group; however, this difference did not reach statistical significance. These findings suggest that PT may provide acceptable disease control during the first postoperative year in selected patients with primary sacrococcygeal PSD. However, the study was not designed as an equivalence or non-inferiority trial, and the nonsignificant difference in recurrence rates should not be interpreted as proof of equivalence between the 2 techniques. Moreover, because PSD may recur late, longer follow-up is required to determine the long-term durability of both treatment modalities.

Postoperative complications were observed only in the KF group in the present study. Seroma and surgical site infection were the recorded complications, both of which are recognized postoperative problems after flap-based surgical procedures for PSD. These complications were managed with percutaneous needle aspiration for seroma and local wound care with oral antibiotic therapy for surgical site infection. Importantly, none of the patients required reoperation. In contrast, no postoperative complications were observed in the PT group, which may be related to the absence of wide excision, flap mobilization, and suture-line tension. This finding supports the potential advantage of PT in reducing early postoperative morbidity in selected patients.

An important issue in the interpretation of these findings is patient selection. Although the baseline demographic characteristics were similar between the 2 groups, disease-specific severity parameters such as the number of sinus openings, size of the sinus cavity, extent of lateral tracts, and distance from the midline were not systematically available in the retrospective records. Therefore, direct comparison of disease complexity between the groups could not be performed. Accurate disease characterization is also important because rare atypical presentations of PSD, such as perianal localization, may mimic other conditions and complicate differential diagnosis.^[29]^ This limitation should be considered when interpreting the comparative outcomes, because more extensive disease may influence wound healing, postoperative morbidity, and recurrence. Future prospective studies should include standardized disease severity parameters to better define which patients are most suitable for PT or KF.

The present study has several limitations. First, its retrospective design may have introduced selection bias, particularly because treatment modality was selected after discussion with the patient rather than by randomization. Second, the unequal group sizes, with 77 patients in the KF group and 48 patients in the PT group, may have reduced the statistical power to detect differences in some outcomes, especially recurrence. A post hoc power assessment was performed for the comparison of recurrence rates between the PT and KF groups. Based on the observed recurrence rates of 12.5% in the PT group and 9.1% in the KF group, with a 2-sided alpha level of 0.05 and the current sample sizes, the estimated statistical power was approximately 9%. This low statistical power indicates that the study may have been underpowered to detect a small difference in recurrence rates and supports the possibility of a type II error. Therefore, the nonsignificant difference in recurrence rates should not be interpreted as definitive equivalence between the 2 techniques. Third, although all included patients completed 1-year follow-up, part of the follow-up assessment was based on structured telephone interviews. Asymptomatic recurrence may therefore have been missed in patients who did not undergo physical examination. Finally, the 1-year follow-up period may be insufficient to fully evaluate late recurrence in PSD.

Despite these limitations, the present study has several strengths. It directly compared a minimally invasive treatment modality with an established off-midline flap procedure in patients with primary sacrococcygeal PSD. In addition to recurrence, clinically relevant outcomes such as wound healing time, return-to-work time, and postoperative complications were evaluated. These outcomes are particularly important in young and working-age patients, in whom treatment success is not determined only by recurrence but also by the speed of recovery and the ability to return to daily life.

Overall, the findings of the present study suggest that PT may be considered a practical minimally invasive alternative to KF in selected patients with primary sacrococcygeal PSD. KF remains an effective surgical option with acceptable recurrence rates, particularly when flap-based treatment is preferred or required. However, PT may offer important advantages in terms of faster wound healing, earlier return to work, and lower early postoperative morbidity. Treatment selection should therefore be individualized according to disease characteristics, patient preference, and the relative importance of rapid postoperative recovery.

## 5. Conclusion

In patients with primary sacrococcygeal PSD, PT was associated with shorter wound healing and return-to-work times and lower early postoperative morbidity compared with KF. The 1-year recurrence rate was numerically higher in the PT group, but the difference was not statistically significant. These findings suggest that PT may be considered a practical minimally invasive alternative in selected patients, particularly when rapid recovery and early return to daily activities are important treatment goals. However, prospective randomized studies with larger sample sizes, standardized disease severity assessment, and longer follow-up are needed to better define the optimal indications for PT and KF.

## Author contributions

**Conceptualization:** İgbal Osmanov, Uğur Ekici.

**Data curation:** İgbal Osmanov, Bariş Hazir.

**Formal analysis:** İgbal Osmanov.

**Investigation:** İgbal Osmanov, Bariş Hazir.

**Methodology:** İgbal Osmanov, Uğur Ekici.

**Supervision:** Uğur Ekici.

**Writing – original draft:** İgbal Osmanov.

**Writing – review & editing:** İgbal Osmanov, Bariş Hazir, Uğur Ekici.
